# Body Posture Defects and Body Composition in School-Age Children

**DOI:** 10.3390/children7110204

**Published:** 2020-10-29

**Authors:** Jacek Wilczyński, Magdalena Lipińska-Stańczak, Igor Wilczyński

**Affiliations:** Laboratory of Posturology, Collegium Medicum, Jan Kochanowski University in Kielce, 25-369 Kielce, Poland; magdal.mail@wp.pl (M.L.-S.); wilczynskii@o2.pl (I.W.)

**Keywords:** body posture defects, body composition, school-children

## Abstract

The aim of the study was to assess the relationship between the shape of the anteriorposterior spinal curvature and body composition in schoolchildren. The study included 257 children, aged 11–12. Correct spinal curvature was established in 106 (41.08%) subjects. Other types included: decreased kyphosis and correct lordosis—40 participants (15.50%), correct kyphosis and decreased lordosis—24 individuals (9.30%), increased kyphosis and correct lordosis—17 subjects (6.59%), correct kyphosis and increased lordosis—22 children (8.53%), decreased kyphosis and decreased lordosis—32 people (12.40%), decreased kyphois and increased lordosis—four of the examined subjects (1.55%) increased kyphosis and lordosis—13 people (5.04%). In addition, 134 (51.94%) demonstrated scoliotic posture and eight (3.10%) scoliosis. There were significant relationships between the shape of the anteriorposterior curvatures and body composition in schoolchildren. Those with a strong body build (predominance of mesomorphs) were generally characterised by the correct formation of these curvatures. In contrast, lean subjects (with the predominance of ectomorphic factors) were more likely to experience abnormalities. No correlations with body composition were observed in the group with scoliotic posture or scoliosis. Both in the prevention and correction of postural defects, one should gradually move away from one-sided, usually one-system, therapeutic effects. An approach that takes into account both somatic and neurophysiological factors seems appropriate. With the correct body composition and structure, shaping the habit of correct posture is much easier.

## 1. Introduction

Postural defects constitute a significant health problem. Despite the many methods of diagnosis, the exact cause has not yet been established. Therefore, it is very important to pay attention to all the conditions that play a significant role in postural development. Our research was further inspired by the unsatisfactory effects of postural re-education. Postural defects and scoliosis are the result of genetic and environmental factors. The level of physical activity, type of body build and composition also play an indirect role [1]. These disadvantages are wrongly considered a local problem. Although their general impact on the entire motor apparatus and gait is emphasized, their therapy is still usually limited to local correction. Body posture is a psychomotor habit that is related to somatic development, body composition and structure. Posture is not a static set of body segments, but is a highly automated movement act. Its health importance is most often emphasized by influencing the arrangement and functions of internal systems and organs. It is also a factor affecting the stability and balance of the body. Economical energy expenditure also depends on correct posture. In addition, it has significant impact on emotional and cognitive spheres, including the development of a child’s speech. Correct posture is fundamental in school education. When the postural system functions smoothly, the child is free to focus on learning rather than dealing with the meticulous control of her/his posture. Therefore, taking care of correct posture goes far beyond the aesthetic sphere [2].

Body posture, as a chain of numerous conditional and unconditional reflexes, is essentially a dynamic stereotype, a kind of movement habit. The final determination of the anterior-posterior spinal curvature in overcoming gravity is determined by sets of conditional reflexes and movement habits. They are created during the period of development on the basis of unconditional postural, positional, support, static-kinetic and motion-based reflexes [3]. Factors from the external environment also play an important role in the development of posture. This happens through the development of increasingly higher forms of functional adaptation of body posture and movement to the outside world [3]. Environmental influences are responsible for developmental variability, intra-individual variability and, as a result of inter-individual diversity, individuality in terms of body posture. Therefore, human body posture is similar for species, but developmentally variable and individually differentiated. Body posture of a child who has just achieved a standing position, therefore differs from his/her posture in subsequent periods of development. However, this is always an individual characteristic, to the extent that it is often a criterion for recognising a given person [4].

Before the final shaping of posture, appropriate mechanisms conditioning the ability to resist gravity must work. Here, a specialised gravitational system plays a coordinating role. The correct posture of the body is an integrated system of osteoarticular and fascial-ligamentous-muscular structures controlled by the central nervous system to ensure optimal conditions for development and puberty [5].

Any deviations in the operation of this system should be treated as defects requiring correction on the basis of feedback and self-control or reconstruction, enabling the development of more favourable stato-kinetic patterns. Hence, the criteria for correct posture cannot be constant and unambiguous for everyone; on the contrary, they should undergo changes depending on the child’s developmental period. In this assessment, factors such as constitutional type, sports disciplines and forms of recreation cannot be ignored [6].

The formation of anterior-posterior spinal curvatures and body posture depend on both genetic determinants and interaction betweenthe nervous and endocrine systems. Hence, posture defects may have their cause in both nervous system dysfunctions, hormonal disorders or in developmental and post-traumatic deformations of the trunk or limbs [7].

Changes in posture and the shape of anterior-posterior spinal curvatures that occur during the growth of children are one of the manifestations of somatic development. However, the concept of body posture should be distinguished from its build. Admittedly, both properties, posture and build, are an expression of special conditions related to the osteoarticular and fascial-ligamentous-muscular system. They create an image of the body’s spatial arrangement, mainly the locomotor system, but are based on different mechanisms [8].

Body structure basically depends on somatic structure and body composition. Body posture, as a chain of numerous conditional and unconditional reflexes, is essentially a dynamic stereotype, a kind of movement habit. It is based on the neurophysiological function conditioning the state of proper tension in appropriate muscle groups. This leads to one and no other arrangement of individual body segments relative to each other, determining overall balance [9].

The aim of the study was to assess the relationship between the shape of anterior-posterior spinal curvatures and body composition in schoolchildren.

## 2. Material and Methods

The study included 257 children aged 11–12 qualified for examination. The children were divided into groups according to age and sex. There were 66 girls aged 11, 67 girls aged 12, 70 boys aged 11 and 60 boys aged 12. Research was conducted from December 2016 to the end of May 2017 at the Posturology Laboratory of the Jan Kochanowski University (UJK) in Kielce. Research was conducted with the consent of the Bioethical Committee of Jan Kochanowski University in Kielce, No. 20/2015. We have obtained consent for publication from the legal guardians of all the participants with individual patient data. The subjects were randomly selected after prior determination of the criteria to be met by individual groups. Before beginning the study, the following documents were analysed: information on the subject containing detailed data on the purpose and rules of conducting the study; consent form of the parent/legal guardian for the child’s participation; declaration of the parent/legal guardian of the child for the processing of data related to participation in the study; declaration, signed with my name and surname, giving informed consent for the examination from all parents/legal guardians of children. Each person could refuse to participate in the research or withdraw at any time—also during the study, without suffering any consequences. The inclusion criteria were: age 11–12 years, no certificate of physical or intellectual disability, no diagnosed syndromes or congenital defects of the CNS or locomotor system, preventing proper psychomotor development, no genetic syndromes, hormonal disorders, neuromuscular diseases, no congenital motor system defects, written consent of parents or guardians for testing. The exclusion criteria were: the presence of syndromes and congenital defects of the CNS or the musculoskeletal system, preventing proper psychomotor development, a certificate of physical or intellectual disability, disorders that may be the cause of pathological body posture: genetic syndromes, hormonal disorders, neuromuscular diseases, congenital defects of the locomotor system, age below 10 and above 11, no written consent for testing. Children took part in curricular physical education classes three times a week. The vast majority did not engage in competitive sports at sports clubs.

Examination of body posture and the spine began with clinical examination. Visual assessment of posture (symmetry test), the spine as well as the e-bend test (Adam’s test) were performed, and the length of the lower limbs was also measured [10]. Body posture and the spine were examined with the Diers Formetric III 4D optoelectronic method. Three-dimensional analysis of body posture and the spine is a combination of digital data processing and the latest optical imaging technique. The test enables non-contact and fast 4D photogrammetric measurement. The measurement results are very precise, and thanks to sending the image to a computer, data analysis takes place immediately after the test. At a distance of about 3 m from the optical tripod, a dark background was mounted. During the measurement, the subject was positioned with his/her back to the camera at a distance of 2 m. S/he assumed normal posture, with his/her feet placed in front of the marked line. The projector emitted horizontal stripes about 1-cm wide onto the subject’s back. As recommended by the manufacturer of the Diers Formetric III 4D, examination of body posture and spine was carried out with DiCAM program using the “Average” measurement option. This consisted of taking a sequence of 12 film frames. Then, by creating an average value, the variance of posture was reduced, thus, improving the value of the study. Then the computer program data were analysed, and a digital, photogrammetric image of the spine was created. The test was performed twice. The researcher decided which trial more adequately reflected actual posture, and only this examination was further analysed. The measurements were performed before noon. Body posture testing with the Diers Formetric III 4D device lasted about 15 min [11]. Spinal measurements were performed from C_7_ to S_1_ the following parameters describing the child’s posture were analysed: kyphotic angle (°), lordotic angle (°), pelvic tilt (mm), lateral deviation (mm) and surface rotation (°). The norms for kyphotic and lordotic angles, on the basis of which body posture in the sagittal plane was assessed, are: kyphotic angle 47–50°, lordotic angle 38–42°. On their basis, 8 spinal types were distinguished in the sagittal plane: correct curvature of the spine (kyphotic angle 47–50°, lordotic angle 38–42°); decreased kyphosis and correct lordosis (kyphotic angle < 47°, lordotic angle 38–42°); correct kyphosis and decreased lordosis (kyphotic angle 47–50°, lordotic angle < 42°); increased kyphosis and correct lordosis (kyphotic angle > 50°, lordotic angle 38–42°); correct kyphosis and increased lordosis (kyphotic angle 47–50°, lordotic angle > 42°); decreased kyphosis and decreased lordosis (kyphotic angle < 47°, lordotic angle < 42°); decreased kyphosis and increased lordosis (kyphotic angle < 47°, lordotic angle > 42°); increased kyphosis and increased lordosis (kyphotic angle > 50°, lordotic angle > 42°) [12]. The occurrence of scoliotic posture and scoliosis was found by considering the values of 3 variables: pelvic tilt (mm), lateral deviation (mm) and surface rotation (°). The following division was adopted for assessment: scoliotic posture: pelvic tilt less than 5 mm, lateral deviation (rms) less than 5 mm, rotation (rms) less than 5°; scoliosis: pelvic tilt equal to or greater than 5 mm, lateral deviation (rms) equal to or greater than 5 mm, rotation (rms) equal to or greater than 5°. To assess the presence of scoliotic posture or scoliosis, all 3 conditions had to be met. If 3 requirements were not met, it was assumed that scoliosis did not occur.

Body composition was measured using Bioelectrical impedance analysis (BIA) with the Tanita MC-780 multi-frequency segment body composition analyser. This non-invasive testing method allows analysis of body composition using the electrical resistance of the body’s tissues, so-called impedance. The Tanita MC-780 segment body composition analyser applies innovative Multi Frequency technology, i.e., currents with variable frequencies: 5, 50, 250 and 500 kHz. This made it possible to assess resistance and conductivity of the tissues. The flow of alternating currents was possible due to different fluid contents in the tissues. Body composition analysis of individual segments (upper and lower limbs, trunk) was conducted taking body side (right and left) and the distinction between tissue reactance and resistance (muscle, fat and visceral tissue) into account. The patient was examined in a standing position, the feet (placed on the base of the analyser) in contact with the built-in electrodes. Patient data (age, gender, body height) were entered by the investigator. During the first stage, body mass was determined, at the next stage (patient’s hands—on the handles with built-in electrodes), impedance was measured. Full segment analysis was carried out in 30 s. Values of segment measurements for the lower and upper limbs as well as the trunk are expressed in specific SI units: (kg), (kJ), (%), (°), (Ω), (kg/m^2^). The body analyser had a certificate confirming its potential application in medical fields. The following variables were analysed in the study: body mass (kg), basal metabolic rate (BMR) (kJ), fat percentage (FATP) (%), fat mass (FATM) (kg), fat-free mass (FFM) (kg), total body water (TBW) (kg), predicted muscle mass (PMM) (kg), body mass index (BMI) (kg/m^2^), visceral fat level (VFATL), bone mass (BONEM) (kg), extracellular water (ECW) (kg), intracellular water (ICW) (kg), proteins (kg) and metabolic age (METAAGE) (years).

The results of the survey were summarised using the PQStat version 1.6.4.121 statistical package. Normality of distribution regarding body posture and composition variables were assessed using the Kolmogorov–Smirnov test. Differences between variables according to sex and age of the subjects were compared by the Mann–Whitney U test. Analysis of the relationship between body posture defects and composition was carried out via the Kruskal–Wallis test and the Dunn–Bonferroni post-hoc test. The test probability of *p* < 0.05 was considered statistically significant.

## 3. Results

**Figure 1:**
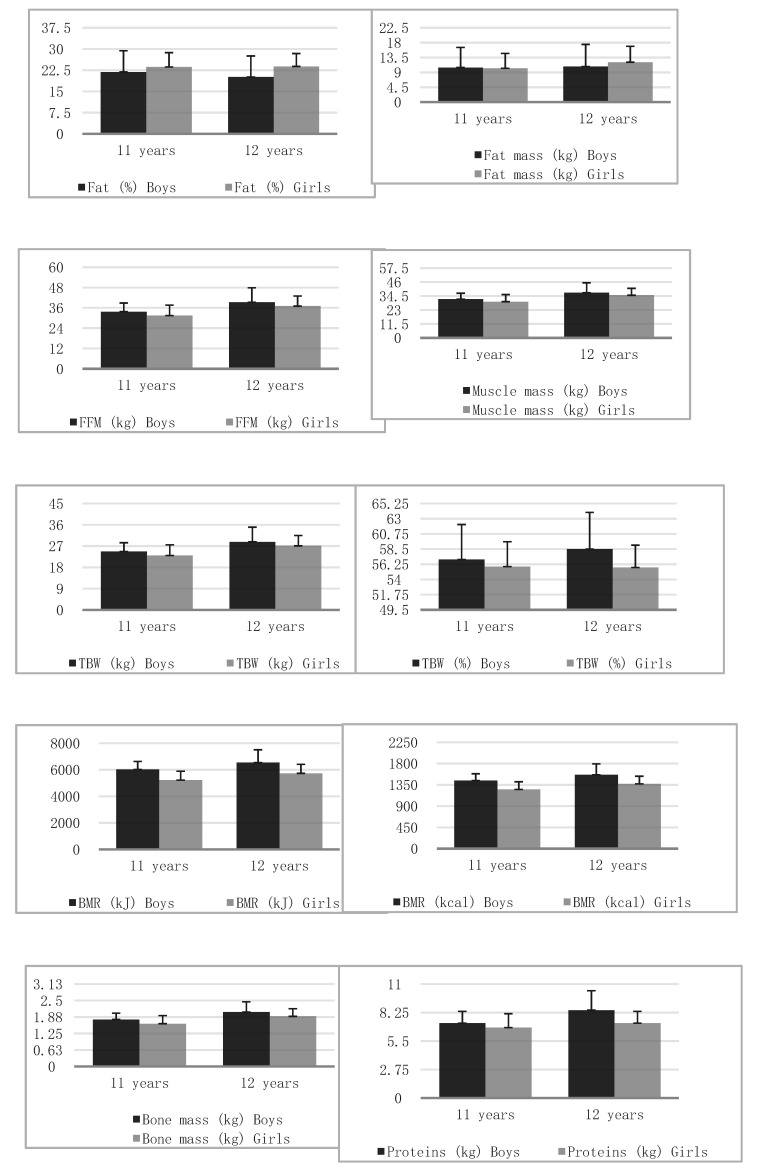
Bar plots illustrating the mean and standard deviation of analyzed variables in groups of boys and girls.

Correct spinal curvature was noted in 106 (41.08%) persons. The remaining types were: decreased kyphosis and correct lordosis—40 people (15.50%), correct kyphosis and decreased lordosis—24 individuals (9.30%), increased kyphosis and correct lordosis, 17 subjects (6.59%), correct kyphosis and increased lordosis, 22 people (8.53%), decreased kyphosis and decreased lordosis, 32 patients (12.40%), decreased kyphosis and increased lordosis, four people (1.55%), increased kyphosis and increased lordosis, 13 participants (5.04%). In addition, 134 (51.94%) subjects demonstrated scoliotic posture and eight (3.10%), scoliosis. Significant differences were observed in the vast majority of body composition variables between girls and boys (Table 1, Figure 1).

Body composition results differed significantly (*p* < 0.05) depending on the type of spine. Body height showed significant (*p* = 0.041) differences regarding spine type in post-hoc analyses, nonetheless, no specific differences between the groups could be indicated (*p* > 0.05). Body mass showed highly significant (*p* = 0.0018) differences depending on spine type and in post-hoc analyses, a significant difference (*p* = 0.0230) concerned the comparison of the groups “decreased kyphosis and increased lordosis” and “increased kyphosis and increased lordosis”. Body height (cm) in the group of 11-year-old boys was at an average of 151.44, with a standard deviation of 5.49. The median distribution of results was 151 and the range of results from 136 to 164. However, among girls, the average was 149.35 with a standard deviation of 8.23. The median distribution of results was 149.5 and the range of results was from 134 to 171. There were no significant differences between the results for both sexes (*p* = 0.0973) (Table 1, Figure 1).

**Figure 2:**
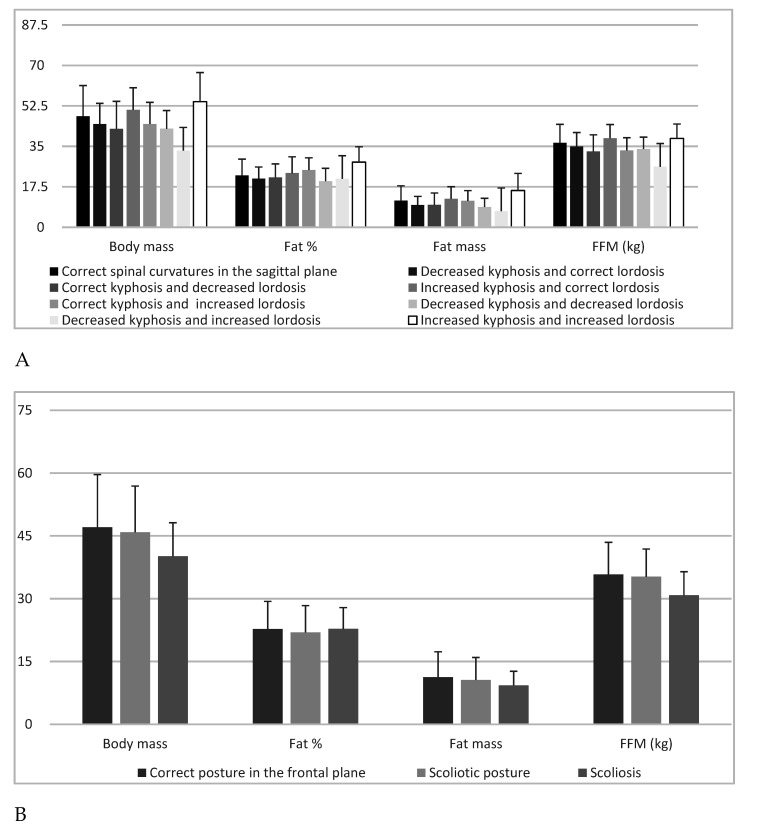
Body mass, fat %, fat mass, fat-free mass and: sagittal spine types (**A**) and correct posture in the frontal plane, scoliotic posture and scoliosis (**B**).

Fat (%) showed highly significant (*p* = 0.005) differences concerning the type of spine in post-hoc analyses, and a significant difference was noted when comparing the groups “increased kyphosis and increased lordosis” with “decreased kyphosis and decreased lordosis” (*p* = 0.003) and “decreased kyphosis and correct lordosis “(*p* = 0.036). Fat mass (kg) showed highly significant (*p* = 0.004) differences depending on spine type in post-hoc analyses. Highly significant (*p* = 0.007) were differences in the comparison of the group “increased kyphosis and increased lordosis” with “decreased kyphosis and decreased lordosis”. FFM (kg) showed highly significant (*p* = 0.002) differences with regard to the defect in the sagittal plane, and in post-hoc analyses, a significant (*p* = 0.033) difference was found when comparing the groups “decreased kyphosis and increased lordosis” with “increased kyphosis and correct lordosis” (Table 2, Figure 2).

**Figure 3:**
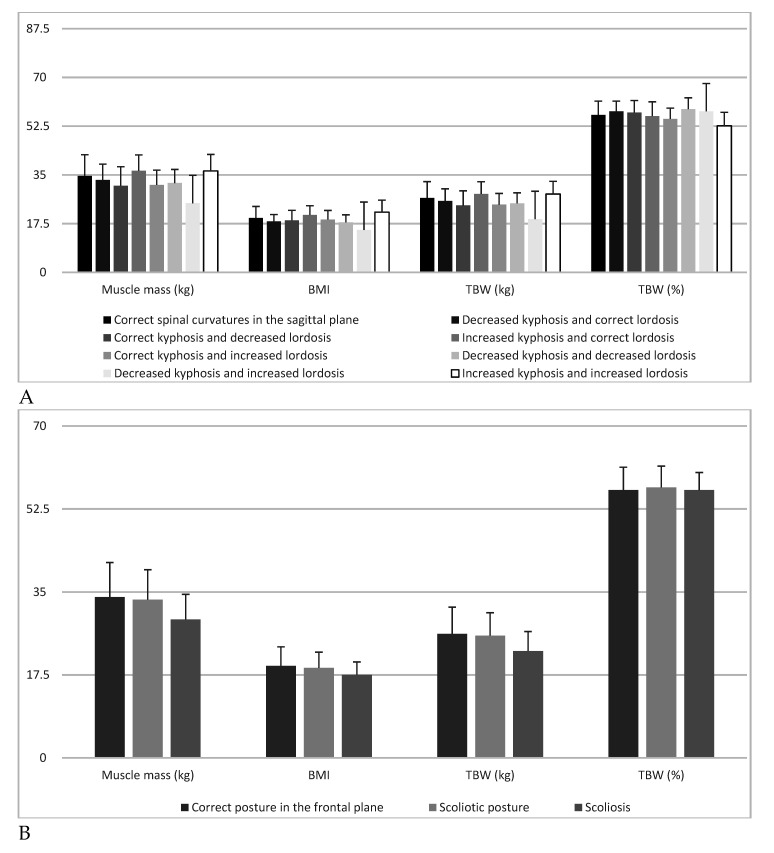
Muscle mass, body mass index, total body water and: sagittal spine types (**A**) and correct posture in the frontal plane, scoliotic posture and scoliosis (**B**).

Muscle mass (kg) exhibited highly significant (*p* = 0.002) differences depending on the type of spine in post-hoc analyses and significant (*p* = 0.033) differences related to the comparison of the group “decreased kyphosis and increased lordosis” with “increased kyphosis and correct lordosis”. BMI demonstrated significant (*p* = 0.010) differences according to the type of spine in post-hoc analyses, however, no specific differences between the groups could be indicated (*p* > 0.05). TBW (kg) demonstrated highly significant (*p* = 0.0029) differences depending on spine type in post-hoc analyses, while a significant (*p* = 0.032) difference was found in the comparison of the group “decreased kyphosis and increased lordosis” with “increased kyphosis and correct lordosis”. TBW (%) showed highly significant (*p* = 0.005) differences with regard to the type of spine, and in post-hoc analyses, significant differences concerned the comparison of the group “increased kyphosis and increased lordosis” with “decreased kyphosis and decreased lordosis” (*p* = 0.003) and “decreased kyphosis and correct lordosis” (*p* = 0.034) (Table 3, Figure 3).

**Figure 4:**
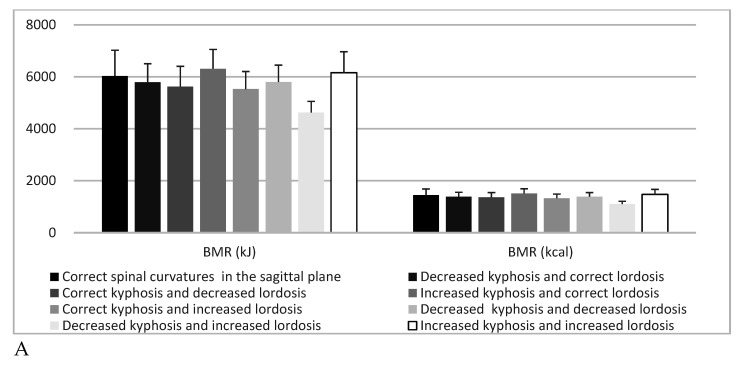

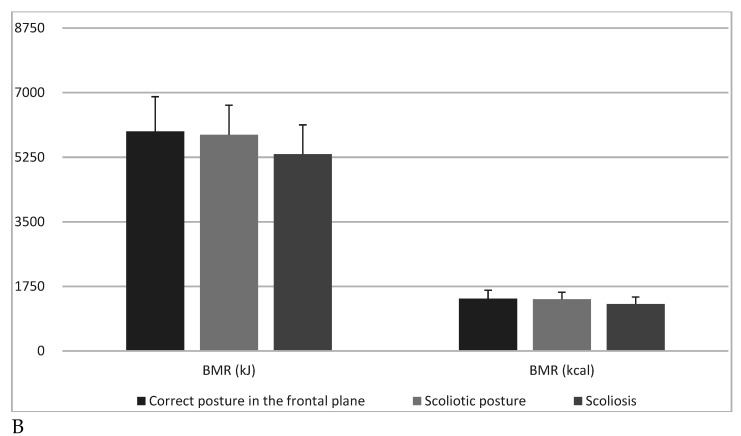
Basal metabolic rate and: sagittal spine types (**A**) and correct posture in the frontal plane, scoliotic posture and scoliosis (**B**).

**Figure 5:**
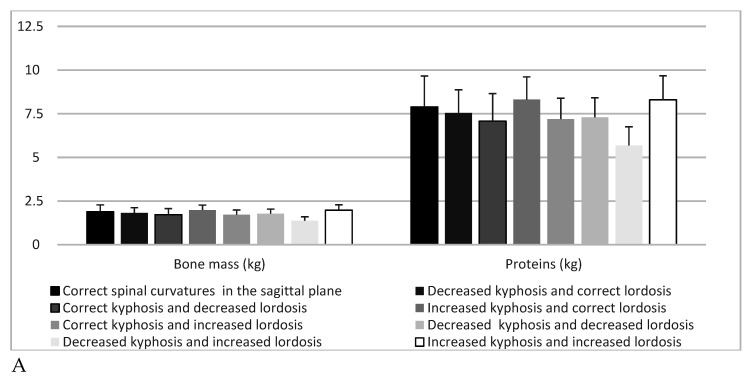

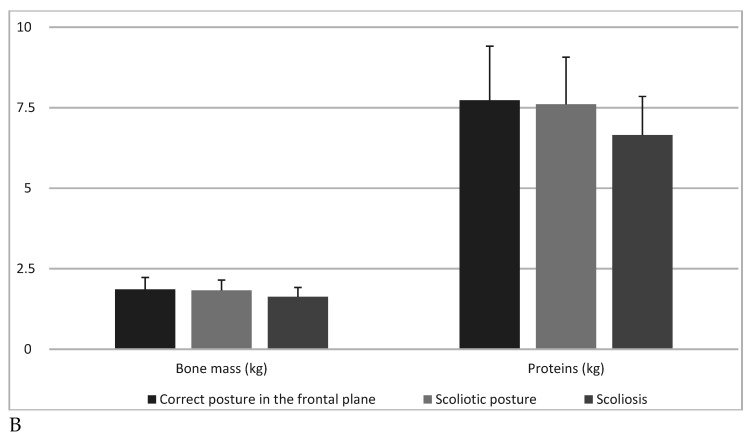
Bone mass, proteins and: sagittal spine types (**A**) and correct posture in the frontal plane, scoliotic posture and scoliosis (**B**).

BMR (kJ) showed highly significant (*p* = 0.001) differences depending on spine type, and in post-hoc analyses, significant differences were found regarding the comparison of the group “decreased kyphosis and increased lordosis” with “correct posture” (*p* = 0.047) and “increased kyphosis and increased lordosis”(*p* = 0.046), as well as “increased kyphosis and correct lordosis” (*p* = 0.005). BMR (kcal) exhibited highly significant (*p* = 0.002) differences according to type of spine in post-hoc analyses, and significant differences applied when comparing the group “decreased kyphosis and increased lordosis” with “correct posture” (*p* = 0.0473), as well as “increased kyphosis and increased lordosis” (*p* = 0.0468) and “increased kyphosis and correct lordosis” (*p* = 0.005). Bone mass (kg) indicated highly significant (*p* = 0.002) differences depending on spine type, and in post-hoc analyses, a significant difference (*p* = 0.018) concerned the comparison of group “decreased kyphosis and increased lordosis” with “increased kyphosis and correct lordosis”. Proteins (kg) showed highly significant (*p* = 0.003) differences depending on the type of spine in post-hoc analyses, the significant difference (*p* = 0.037) highlighted in the comparison of the group “decreased kyphosis and increased lordosis” with “increased kyphosis and correct lordosis”. In contrast, body composition results did not differ significantly (*p* > 0.05) with regard to scoliosis (Table 4, Figure 4 and Figure 5).

## 4. Discussion

Correct posture is a decisive factor in body stability based only on small planes of both feet [13]. It is also a condition for the economic expenditure of energy for body balance, further affecting the proper positioning of internal organs and their functions [14]. Slight adaptation deviations in posture may prove to be beneficial from the point of view of effort-related economics. However, far-reaching adaptations to a given activity are already unfavourable [15]. They lead to defects in posture and, consequently, in body build. Therefore, compensatory procedures are recommended for people who tend to maintain an unchanged position for long periods of time, among others, students spending many hours sitting in front of a school desk [16].

Correct posture is also a body system that provides proper conditions for all body functions, and at the same time, enables active human behaviour towards the environment [17]. This behaviour requires a certain state of alertness, which is associated with greater metabolism and significant energy expenditure [18]. This is true when measuring muscle EMG for correct and improper posture. In correct posture, EMG shows more intensive muscle work [19].

Fatigue, quickly occurring with the involvement of muscles in static efforts, effectively prevents the system by frequent change of position, by relieving one of the muscles, and burdening the others with work [20]. This happens not only while standing, during the phenomenon of so-called deflection, but also in other positions, even while sleeping [21]. Thus, the economic importance of correct posture lies not in the fact that the body is released from effort, but in the fact that it is not exposed to excessive expenditure, which occurs with incorrect body balance [22,23].

Most often, however, the health importance of correct body posture is emphasized. Its relationship with health and the proper functioning of the system is expressed in many forms [24]. Defective formation of anterior-posterior spinal curvatures causes worse ventilation at the top of the lungs, and as a result, a tendency towards respiratory diseases; often leading to head tilt and compression of vessels in the neck region, which impairs cerebral blood supply; lumbar hyperlordosis is the cause of low back pain, neurological disorders and orthostatic proteinuria [25]. With excessive lumbar lordosis, protruding abdomen often occurs. The flaccid abdominal muscles, which do not give proper support to the viscera, lead to incorrect arrangement of organs, mainly the digestive system and abnormal movements of the diaphragm [26]. This weakens digestive and respiratory functions as well as circulation in the abdomen. Disabilities are manifested in digestive disorders, constipation, stabbing sensations, and often irritability and nervousness due to intestinal pressure on nerve plexuses. In girls, the phenomenon of lumbar hyperlordosis sometimes results in menstrual cycle disorders [27,28].

In the authors’ research, there were significant relationships between the formation of sagittal curvatures of the spine and body composition in school-children. People with a strong build (with a predominance of mesomorphs) were generally characterised by correct shape of sagittal spinal curvatures. In contrast, lean people (with the predominance of the ectomorphic factor) were more likely to have abnormalities in curvatures, while body composition results did not differ significantly (*p* > 0.05) depending on scoliosis.

In another, similar study, the authors stated that anthropometry and body composition have plausible influence on paediatric sagittal standing posture. Girls showed increased values of lumbar angle, head and neck flexion, as well as craniocervical angle, with the largest mean (standard deviation) difference in lumbar angle. In both genders, body mass and body mass index were weakly associated with lumbar angle: 0.24  ≥ *r* ≤  0.31 in girls and 0.16 ≥ *r* ≤ 0.26 in boys, for both *p* <  0.001. Fat, fat-free mass and bone mineral density were weakly associated with lumbar angle in both genders. Girls showed increased values of lumbar angle, head and neck flexion, and craniocervical angle, with the largest mean (standard deviation) difference in lumbar angle among boys. In both boys and girls, body mass and body mass index were weakly associated with lumbar angle. Fat and fat-free mass as well as bone mineral density were weakly associated with lumbar angle in both genders [29].

The aim of a different study was to assess the relationship between children’s body mass composition and body posture. The relationship between physical activity level of children and the parameters characterising their posture was also evaluated. The study included 120 school-children between the age of 11 and 13, comprising 61 girls and 59 boys. Each study participant underwent posture evaluation via the photogrammetric method using the projection moiré phenomenon. Moreover, body mass and the level of physical activity were evaluated. Children with the lowest content of muscle tissue showed the highest differences in the height of the inferior angles of the scapulas in the frontal plane. Children with excessive body fat had less curvature of the thoracic-lumbar spine, greater differences in the depth of the inferior angles of the scapula and greater angles of the shoulder line. The individuals with higher levels of physical activity had smaller angle body inclination angles. The content of muscle tissue, adipose tissue, and physical activity level determines the variability of the parameter characterising body posture [30].

The objective of another study was to assess the relationship between body mass index (BMI) and the incidence of abnormalities in selected parameters measured in the trunk area. It was noticed that obese and overweight children tended to assume incorrect positioning of the shoulders and pelvis in comparison to children with normal body mass. It was found that greater body mass (higher BMI) coincided with a larger distance of the scapulae from the frontal plane (*p* = 0.009). An increase in BMI among children causes detrimental effects in scapula positioning, reflected in their greater distance from the frontal plane. The increase in BMI is not significantly correlated with positioning of the shoulder or pelvic joints; however, overweight or obese individuals demonstrated greater differences in their positioning [31].

In another study, the authors aimed to determine which somatic features and parameters of spinal curvatures in the sagittal plane show statistically significant differences among children with given types of body posture. The size-related parameters and indices of anterior-posterior spinal curvatures appeared to be the least differentiating factors among posture types. The strongest similarity of posture types was found in somatic features and weight–height ratios. The size parameters and indices of anterior-posterior spinal curvatures appeared to be the least differentiating factors among posture types. The strongest similarity of posture types was found in somatic features and weight–height ratios [32]. Knowledge regarding the ontogenetic variability of body composition will result in more precise information on the physiological and biochemical processes taking place in the body of a child with postural defects.

A limitation of our research was the lack of body composition analyses conducted in the group of children with severe scoliosis. Postural defects should definitely be distinguished from diseases such as scoliosis. In the etiopathogenetic understanding, scoliosis is merely a symptom, an external expression of an undiagnosed pathology that may appear in a different location in the body and at different ages of a child. Although scoliosis is clearly a deformation of the body, it is also an effect of its compensatory abilities, allowing the child to keep the head and shoulder girdle above the pelvis. The final shape of the trunk is the result of deforming processes and a compensatory reaction, thanks to which the body maintains general orientation of the body at the expense of a great disturbance of its own form. In the present state of knowledge, it is justified to speak about etiological factors, and not about the genetic, metabolic, etc. theory of scoliosis [33]. Currently, the multifactorial concept, including the genetically determined pathology of the central nervous system, has the most supporters. It concerns in particular the broadly understood postural system [34]. This pathology has a long-lasting effect, most likely through the trans-spinal muscle system, on the growing spine with an individually variable and multi-factorial susceptibility to the occurrence of deformity. The progression of the deformity is associated with growth and biomechanical factors. The elucidation of the etiology of scoliosis is the fundamental problem of modern paediatricorthopaedics. A detailed understanding regarding the mechanisms of the early stages of this deformity may indicate the right path to work on the etiology and facilitate the determination of effective methods of prevention and treatment [35].

## 5. Conclusions

Significant relationships were noted between the shape of the anterior-posterior curvatures and body composition in school-children. Individuals with a strong build (predominance of mesomorphs) were generally characterised by the correct formation of these curvatures. In contrast, lean people (with the predominance of the ectomorphic factor) were more likely to have abnormalities within them. In the group of children with scoliotic posture and scoliosis, no relationships with body composition were observed. Both in prophylaxis and postural re-education, one should gradually move away from one-sided, most often one-system, therapeutic effects. An approach that takes into account both somatic and neurophysiological factors seems appropriate. With the correct body composition and structure, developing the habit of correct posture is much easier.

## Figures and Tables

**Table 1:** Results of analysed variables according to gender.

Variables	Age	Boys/Girls	X	SD	Mann–Whitney U Test	Student’s *t*-Test
Body height (cm)	11	Boys	151.44	5.49	Z = 1.877 *p* = 0.060	t = 1.673 *p* = 0.097
		Girls	149.35	8.23		
	12	Boys	158.18	8.71	Z = 0.886 *p* = 0.375	t = −0.768 *p* = 0.444
		Girls	159.24	6.63		
Body mass (kg)	11	Boys	44.19	10.56	Z = 1.420 *p* = 0.155	t = 1.386 *p* = 0.168
		Girls	41.66	10.23		
	12	Boys	50.08	13.72	Z = 0.114 *p* = 0.908	t = 0.446 *p* = 0.656
		Girls	49.11	10.34		
Fat (%)	11	Boys	21.85	7.5	Z = 2.225 *p* = 0.026	t = −1.626 *p* = 0.106
		Girls	23.65	5.06		
	12	Boys	20.1	7.43	Z = 3.993 *p* = 0.000	t = −3.371 *p* = 0.001
		Girls	23.82	4.55		
Fat mass (kg)	11	Boys	10.46	6.07	Z = 0.568 *p* = 0.569	t = 0.245 *p* = 0.806
		Girls	10.23	4.49		
	12	Boys	10.75	6.7	Z = 2.600 *p* = 0.009	t = −1.271 *p* = 0.206
		Girls	12.07	4.81		
FFM (kg)	11	Boys	33.72	5.11	Z = 2.561 *p* = 0.010	t = 2.334 *p* = 0.021
		Girls	31.42	6.16		
	12	Boys	39.33	8.49	Z = 1.333 *p* = 0.182	t = 1.749 *p* = 0.083
		Girls	37.04	5.94		
Muscle mass (kg)	11	Boys	31.9	4.92	Z = 2.467 *p* = 0.0136	t = 2.229 *p* = 0.027
		Girls	29.8	5.85		
	12	Boys	37.28	8.1	Z = 1.288 *p* = 0.197	t = 1.707 *p* = 0.090
		Girls	35.15	5.65		
BMI	11	Boys	19.13	3.76	Z = 0.739 *p* = 0.459	t = 1.046 *p* = 0.297
		Girls	18.48	3.26		
	12	Boys	19.77	4.1	Z = 0.345 *p* = 0.729	t = 0.797 *p* = 0.426
		Girls	19.25	3.28		
TBW (kg)	11	Boys	24.69	3.75	Z = 2.554 *p* = 0.010	t = 2.334 *p* = 0.021
		Girls	23	4.52		
	12	Boys	28.79	6.22	Z = 1.331 *p* = 0.183	t = 1.754 *p* = 0.082
		Girls	27.11	4.35		
TBW (%)	11	Boys	56.96	5.18	Z = 1.965 *p* = 0.049	t = 1.358 *p* = 0.176
		Girls	55.9	3.69		
	12	Boys	58.49	5.45	Z = 4.024 *p* = 0.000	t = 3.375 *p* = 0.001
		Girls	55.77	3.31		
BMR (kJ)	11	Boys	6029.91	597.4	Z = 6.367 *p* < 0.001	t = 7.255 *p* < 0.001
		Girls	5220.55	674.44		
	12	Boys	6538.84	970.67	Z = 5.021 *p* < 0.001	t = 5.393 *p* < 0.001
		Girls	5733.16	678.25		
BMR (kcal)	11	Boys	1441.17	142.78	Z = 6.195 *p* < 0.001	t = 7.036 *p* < 0.001
		Girls	1252.75	162.54		
	12	Boys	1562.82	231.99	Z = 5.021 *p* < 0.001	t = 5.394 *p* < 0.001
		Girls	1370.25	162.11		
Bone mass (kg)	11	Boys	1.78	0.24	Z = 3.580 *p* = 0.001	t = 3.291 *p* = 0.001
		Girls	1.62	0.31		
	12	Boys	2.06	0.39	Z = 2.227 *p* = 0.025	t = 2.588 *p* = 0.010
		Girls	1.9	0.29		
Proteins (kg)	11	Boys	7.23	1.13	Z = 2.270 *p* = 0.023	t = 2.014 *p* = 0.046
		Girls	6.8	1.34		
	12	Boys	8.48	1.88	Z = 1.114 *p* = 0.265	t = 1.550 *p* = 0.124
		Girls	7.23	1.13		

**Table 2:** Body posture defects according to body composition.

Variables	N; %	Body Mass (kg)		Fat (%)		Fat Mass (kg)		FFM (kg)	
		X; SD	Kruskal–Wallis Test	X; SD	Kruskal–Wallis Test	X; SD	Kruskal–Wallis Test	X; SD	Kruskal–Wallis Test
Correct spinal curvatures in the sagittal plane	106% 41.08	48.00 ± 13.29	H = 22.81 *p* = 0.001	22.46 ± 7.02	H = 20.28 *p* = 0.005	11.50 ± 6.42	H = 20.49 *p* = 0.004	36.50 ± 8.03	H = 21.648 *p* = 0.002
Decreased kyphosis and correct lordosis	40% 15.50	44.62 ± 8.98		21.02 ± 5.03		9.62 ± 3.71		35.00 ± 5.97	
Correct kyphosis and decreased lordosis	24% 9.30	42.58 ± 11.89		21.58 ± 5.82		9.75 ± 5.07		32.84 ± 7.14	
Increased kyphosis and correct lordosis	17% 6.59	50.79 ± 9.53		23.42 ± 7.05		12.29 ± 5.24		38.50 ± 5.93	
Correct kyphosis and increased lordosis	22% 8.53	44.64 ± 9.39		24.74 ± 5.29		11.40 ± 4.44		33.24 ± 5.48	
Decreased kyphosis and decreased lordosis	32% 12.40	42.63 ± 7.86		19.94 ± 5.55		8.77 ± 3.75		33.87 ± 5.12	
Decreased kyphosis and increased lordosis	4% 1.55	33.18 ± 6.16		20.93 ± 3.41		6.98 ± 1.79		26.20 ± 4.84	
Increased kyphosis and increased lordosis	13% 5.04	54.31 ± 12.60		28.16 ± 6.62		15.90 ± 7.37		38.41 ± 6.24	
Correct posture in the frontal plane	116% 44.96	47.06 ± 12.58	H = 2.252 *p* = 0.324	22.77 ± 6.58	H = 1.055 *p* = 0.589	11.28 ± 6.04	H = 1.005 *p* = 0.604	35.78 ± 7.67	H = 3.011 *p* = 0.221
Scoliotic posture	134% 51.94	45.86 ± 11.04		21.95 ± 6.40		10.61 ± 5.33		35.25 ± 6.61	
Scoliosis	8% 3.10	40.14 ± 7.99		22.78 ± 5.08		9.31 ± 3.34		30.83 ± 5.60	

**Table 3:** Body posture defects according to body composition.

Variables	N; %	Muscle Mass (kg)		BMI		TBW (kg)		TBW (%)	
		X; SD	Kruskal–Wallis Test	X; SD	Kruskal–Wallis Test	X; SD	Kruskal–Wallis Test	X; SD	Kruskal–Wallis Test
Correct spinal curvatures in the sagittal plane	106% 41.08	34.61 ± 7.65	H = 21.907 *p* = 0.002	19.5 ± 4.08	H = 18.397 *p* = 0.011	26.72 ± 5.88	H = 21.668 *p* = 0.002	56.57 ± 4.91	H = 20.247 *p* = 0.005
Decreased kyphosis and correct lordosis	40% 15.50	33.17 ± 5.69		18.3 ± 2.41		25.62 ± 4.36		57.85 ± 3.63	
Correct kyphosis and decreased lordosis	24% 9.30	31.12 ± 6.80		18.7 ± 3.56		24.05 ± 5.22		57.42 ± 4.28	
Increased kyphosis and correct lordosis	17% 6.59	36.51 ± 5.65		20.6 ± 3.31		28.20 ± 4.35		56.09 ± 5.14	
Correct kyphosis and increased lordosis	22% 8.53	31.39 ± 5.32		18.9 ± 3.26		24.33 ± 4.01		55.10 ± 3.88	
Decreased kyphosis and decreased lordosis	32% 12.40	32.09 ± 4.86		17.9 ± 2.66		24.79 ± 3.76		58.61 ± 4.08	
Decreased kyphosis and increased lordosis	4% 1.55	24.83 ± 4.62		15.2 ± 2.25		19.15 ± 3.55		57.83 ± 2.54	
Increased kyphosis and increased lordosis	13% 5.04	36.43 ± 5.93		21.6 ± 4.30		28.13 ± 4.57		52.62 ± 4.84	
Correct posture in the frontal plane	116% 44.96	33.92 ± 7.30	H = 3.041 *p* = 0.218	19.4 ± 4.01	H = 1.318 *p* = 0.517	26.19 ± 5.62	H = 3.038 *p* = 0.218	56.51 ± 4.80	H = 1.07 *p* = 0.598
Scoliotic posture	134% 51.94	33.40 ± 6.31		19.01 ± 3.31		25.80 ± 4.83		57.04 ± 4.51	
Scoliosis	8% 3.10	29.20 ± 5.31		17.54 ± 2.71		22.55 ± 4.11		56.4 ± 3.72	

**Table 4:** Body posture defects according to body composition.

Variables	N; %	BMR (kJ)		BMR (kcal)		Bone Mass (kg)		Proteins (kg)	
		X; SD	Kruskal–Wallis Test	X; SD	Kruskal–Wallis Test	X; SD	Kruskal–Wallis Test	X; SD	Kruskal–Wallis Test
Correct spinal curvatures in the sagittal plane	106% 41.08	6031.08 ± 992.07	H = 23.04 *p* = 0.001	1441.46 ± 237.11	H = 22.33 *p* = 0.002	1.89 ± 0.39	H = 21.86 *p* = 0.002	7.89 ± 1.77	H = 21.35 *p* = 0.003
Decreased kyphosis and correct lordosis	40% 15.50	5787.25 ± 715.35		1383.18 ± 170.99		1.83 ± 0.29		7.55 ± 1.32	
Correct kyphosis and decreased lordosis	24% 9.30	5625.00 ± 779.26		1356.92 ± 182.16		1.72 ± 0.35		7.08 ± 1.57	
Increased kyphosis and correct lordosis	17% 6.59	6310.00 ± 742.34		1508.1 ± 177.42		1.99 ± 0.28		8.31 ± 1.30	
Correct kyphosis and increased lordosis	22% 8.53	5525.55 ± 675.08		1320.6 ± 161.35		1.72 ± 0.27		7.19 ± 1.20	
Decreased kyphosis and decreased lordosis	32% 12.40	5799.13 ± 649.85		1386.0 ± 155.28		1.78 ± 0.26		7.30 ± 1.11	
Decreased kyphosis and increased lordosis	4% 1.55	4620.00 ± 433.80		1104.2 ± 103.66		1.38 ± 0.22		5.68 ± 1.07	
Increased kyphosis and increased lordosis	13% 5.04	6155.08 ± 807.30		1471.08 ± 192.93		1.98 ± 0.31		8.30 ± 1.37	
Correct posture in the frontal plane	116% 44.96	5948.72 ± 941.73	H = 2.807 *p* = 0.245	1421.78 ± 225.08	H = 2.755 *p* = 0.252	1.86 ± 0.37	H = 2.853 *p* = 0.240	7.73 ± 1.68	H = 3.065 *p* = 0.215
Scoliotic posture	134% 51.94	5860.98 ± 800.24		1403.06 ± 189.95		1.83 ± 0.32		7.61 ± 1.46	
Scoliosis	8% 3.10	5333.00 ± 792.07		1274.63 ± 189.31		1.63 ± 0.29		6.65 ± 1.20	
